# Acute inflammation induced by the *Escherichia coli* lipopolysaccharide considerably increases the systemic and brain exposure of olanzapine after oral administration in mice

**DOI:** 10.1093/ijnp/pyaf036

**Published:** 2025-05-24

**Authors:** Jan Hubeňák, Martin Mžik, Hana Laštůvková, David Bayer, Lenka Jandová, Jolana Schreiberová, Ctirad Andrýs, Stanislav Mičuda, Jiří Masopust, Jaroslav Chládek

**Affiliations:** Department of Psychiatry, University Hospital and the Faculty of Medicine, Charles University, Hradec Kralove, Czech Republic; Department of Clinical Biochemistry and Diagnostics, University Hospital and the Faculty of Medicine, Charles University, Hradec Kralove, Czech Republic; Department of Pharmacology, Faculty of Medicine, Charles University, Hradec Kralove, Czech Republic; Department of Psychiatry, University Hospital and the Faculty of Medicine, Charles University, Hradec Kralove, Czech Republic; Faculty Vivarium, Faculty of Medicine, Charles University, Hradec Kralove, Czech Republic; Department of Pharmacology, Faculty of Medicine, Charles University, Hradec Kralove, Czech Republic; Department of Clinical Immunology and Allergology, University Hospital and the Faculty of Medicine, Charles University, Hradec Kralove, Czech Republic; Department of Pharmacology, Faculty of Medicine, Charles University, Hradec Kralove, Czech Republic; Department of Psychiatry, University Hospital and the Faculty of Medicine, Charles University, Hradec Kralove, Czech Republic; Department of Pharmacology, Faculty of Medicine, Charles University, Hradec Kralove, Czech Republic

**Keywords:** endotoxemia, lipopolysaccharide, olanzapine, pharmacokinetics, brain penetration

## Abstract

**Background:**

A detailed understanding of alterations in olanzapine pharmacokinetics during acute inflammatory states, associated with infections, remains lacking. This study aimed to investigate the impact of endotoxemia on the pharmacokinetics of olanzapine and desmethylolanzapine (DMO) in mice.

**Methods:**

C57BL/6N mice received an intraperitoneal injection of lipopolysaccharide (LPS, 5 mg/kg) or saline (controls), followed 24 hours later by single oral or intravenous doses of olanzapine or intravenous DMO. Concentrations and unbound fractions of olanzapine and DMO were measured in the plasma and brain homogenates.

**Results:**

In LPS-injected mice, the area under the concentration–time curve (AUCs) for olanzapine increased 3.8-fold in the plasma and 5.2-fold in brain homogenates, in consequence of a higher absolute bioavailability of olanzapine (+200%), a lower plasma clearance (−34%), and a higher brain penetration ratio for the unbound drug relative to controls (*K*_p,uu,brain_ 6.2 vs. 4.1). LPS attenuated the hepatic mRNA expression of cytochrome P450 1A2 and the metabolism of olanzapine to DMO. However, the AUC of plasma DMO increased by 140% due to a 4.8-fold decrease in the plasma clearance of DMO. The brain penetration of DMO was minimal (*K*_p,uu,brain_ ≤ 0.051). The LPS-injected mice exhibited a downregulation of the hepatic and ileal mRNA expression of P-glycoprotein (Abcb1a), whereas the expression of Abcb1a and Abcb1b in the brain was upregulated.

**Conclusions:**

Endotoxemia notably increases olanzapine concentrations in the plasma and brain following oral administration in mice. Further studies should clarify whether altered pharmacokinetics results in adverse effects in acutely infected patients taking oral olanzapine.

**Significance Statement**

Individualized dosing of oral olanzapine to patients with schizophrenia and bipolar disorders, supported by therapeutic drug monitoring, is advocated due to its high pharmacokinetic variability. While numerous studies have investigated the underlying factors contributing to this variability, little is known about alterations in olanzapine pharmacokinetics during acute inflammatory responses triggered by infections. We demonstrate that lipopolysaccharide-induced endotoxemia significantly increases systemic and central nervous system (CNS) concentrations of olanzapine in mice dosed with oral olanzapine. This effect is attributed to an increase in bioavailability and CNS penetration, along with a reduction in the first-pass metabolism and metabolic clearance of olanzapine. Despite the suppression of olanzapine metabolism to desmethylolanzapine, endotoxemia elevates the metabolite concentrations and the metabolite-to-parent ratio of concentrations in the plasma due to impaired metabolite elimination. Compared to olanzapine, desmethylolanzapine exhibits minimal CNS penetration. The pronounced impact of endotoxemia on olanzapine pharmacokinetics in mice warrants further investigation in patients.

## INTRODUCTION

Olanzapine, a second-generation antipsychotic drug from the multiacting receptor-targeted antipsychotics (MARTA) group, is widely used to treat schizophrenia and bipolar disorders due to its distinctly favorable efficacy profile. However, long-term therapy is frequently complicated by metabolic syndrome, which is characterized by weight gain, dyslipidemia, and insulin resistance, whereas the risk is lower of extrapyramidal symptoms and hyperprolactinemia.^1^ Acute adverse effects include sedation, hypotension, tachycardia, dizziness, akathisia, and tremors. Numerous clinical pharmacokinetic studies have described significant interindividual variabilities in olanzapine pharmacokinetics, which strongly limits the predictability of the plasma concentrations in individual patients receiving standard therapeutic doses. The average intraindividual variability in the plasma concentrations is one-third to one-half of the interindividual variability.^2-4^ The plasma concentrations of olanzapine correlate well with its concentration in the central nervous system (CNS).^5,6^ These characteristics provide a strong rationale for the pharmacokinetic approach to individualized olanzapine dosing, by utilizing therapeutic drug monitoring to achieve a target plasma through a concentration range of between 20 and 80 ng/mL.^7^ Dose reduction guided by a lower therapeutic range of 20–40 ng/mL has been suggested in the case of a favorable therapeutic response but poor drug tolerance.^8^

Olanzapine undergoes extensive metabolism through parallel and sequential pathways, with the renal and fecal excretion of metabolites. Less than 10% of the bioavailable dose is excreted as unmetabolized olanzapine via the kidneys. In humans, the primary metabolites include N-glucuronides and 4’-N-desmethylolanzapine (DMO), which are predominantly formed in the liver via UDP-glucuronosyltransferase 1A4 (UGT1A4) and cytochrome P450 1A2 (CYP1A2).^9,10^ The hepatic expression of these enzymes varies significantly by up to 40- to 60-fold between individuals and their activity is sensitive to modulation by inhibitors and inducers. Consequently, the large pharmacokinetic variability of oral olanzapine is attributed to factors influencing hepatic clearance, absorption, and first-pass elimination. Population pharmacokinetic studies have identified and quantified the effects of several covariates on olanzapine oral clearance, including smoking, gender, age, body weight (mainly in the pediatric populations), ethnic/genetic variabilities, and pharmacokinetic interactions.^11-13^

Recently, acute inflammatory responses triggered by infections were identified as significant factors influencing the pharmacokinetics (PK) of olanzapine and other antipsychotics.^14^ Pro-inflammatory cytokines, such as interleukin-1 (IL-1), interleukin-6 (IL-6), and tumor necrosis factor-α (TNFα), released during the acute-phase reaction, suppress the phase I and phase II drug-metabolizing enzymes, including CYP1A2 and UGTs, as well as drug transporters. This suppression results in decelerated drug metabolism and altered drug absorption, distribution, and excretion.^15,16^ Increased concentrations and adverse effects of clozapine—a close structural congener of olanzapine metabolized primarily by CYP1A2 to norclozapine—have been documented in patients experiencing acute bacterial infection accompanied by fever, C-reactive protein elevation, and significant cytokine release.^17-19^ Similarly, a recent population pharmacokinetic study reported a 25% reduction in olanzapine oral clearance during infections.^20^ Despite the frequent occurrence of acute inflammatory states and their potential impact on patients being treated with olanzapine, detailed insights into the mechanisms and extent of alterations in olanzapine pharmacokinetics during such conditions remain lacking.

We hypothesized that the systemic inflammatory responses to the infections alter the metabolism and distribution of olanzapine, leading to increased systemic and CNS exposures to the drug. To test this hypothesis, we utilized an endotoxemia model in C57BL/6N mice induced by injecting them with *Escherichia coli* lipopolysaccharides (LPS). The study aimed to characterize changes in the plasma and brain concentrations of olanzapine following a single oral administration. Additionally, we investigated the effects of endotoxemia on olanzapine binding in the plasma and CNS, as well as on the mRNA expression patterns of the key enzymes and membrane transporters involved in its pharmacokinetics.

## METHODS

### Animals

All of the animal experiments were performed in compliance with the EU Directive 2010/63/EU and the animals received care according to the guidelines established by the Animal Welfare Body of the Faculty of Medicine in Hradec Kralove. The project was approved by the Animal Welfare Body of the Ministry of Education, Youth, and Sports (approval No. MSMT-11766/2018-2). Sixteen-week-old male C57BL/6 mice were obtained from Velaz and housed individually in ventilated cages at a constant temperature of 23°C ± 1°C at a humidity of 55% ± 10% under a 12-hour light/dark cycle. The mice had free access to food and water.

### Drug Administration and Sample Collections

After a 1-week acclimation period, the mice were randomly assigned to 2 experimental groups of 36 animals each: (1) an LPS group was given a single 4 mg/kg intraperitoneal (IP) injection of lipopolysaccharide (LPS), serotype O55:B05 (Sigma) and (2) a control group received a vehicle (sterile phosphate-buffered saline). After 24 hours, 18 animals from the LPS group and 18 controls were given a single oral (PO) bolus dose of 4 mg/kg of olanzapine via an oral gavage of 5 mL/kg. The volume load for mice weighing 25 g was 125 µL. This dose was selected based on a previous investigation of olanzapine pharmacokinetics in mice and therapeutic concentrations of olanzapine in patients.^7,21^ Olanzapine powder was dissolved into dimethyl sulfoxide (DMSO), and then diluted by 0.5% (m:v) methyl cellulose (MC) to final concentrations of 0.8 mg/mL olanzapine and 5% dimethylsulfoxide (v:v). The doses of DMSO and MC were 276 and 25 mg/kg. Blood samples from 3 mice for each sampling interval were taken at 0 hour (predose) and 0.25, 0.5, 1, 2, 3, 4, 5, 6, 8, 10, and 24 hours following PO olanzapine. Sampling was performed at 2 intervals in each of the 18 mice injected with LPS and 18 controls. The first blood volume of 150 µL was taken into an EDTA-microvette CB 300 (Sardstedt) from a drop of blood after a small incision at the end of the tail. At the termination interval, the second blood sample was collected by cardiac puncture in terminal anesthesia, and then the animal was put to death by decapitation. Another 20 mice injected with LPS and 20 controls received an intravenous (IV) bolus dose of either 0.5 mg/kg olanzapine (10 animals in both groups) or 0.5 mg/kg DMO (10 animals in both groups). The dose of IV olanzapine was reduced because the bioavailability of PO olanzapine is incomplete and initial concentrations in the plasma are high after a rapid IV injection of the drug. No study has yet addressed the pharmacokinetics of IV DMO. Therefore, the same dose was injected as for IV olanzapine. According to the manufacturer’s instructions, the content of the vial with olanzapine ZYPREXA IM 10 mg powder for injection (Lilly S.A.) was first dissolved with water for injections, and sterile saline was added to dilute the solution 10-fold to a final concentration of 0.5 mg/mL olanzapine. The injected volume was 1 mL/kg, that is, 25 µL for a 25 g mouse. The solution of 0.2 mg/mL DMO (N-desmethyl-olanzapine, Cayman Chemical) was prepared in 1:9 DMSO:phosphate-buffered saline (v:v, pH 7.2). The injected volume was 2.5 mL/kg, that is, 75 µL for a 25 g mouse. After IV administrations, blood was sampled at 3, 5, 10, 20, and 40 minutes, and 1, 1.5, 2, 3, 4, and 6 hours. Blood samples were taken at 3 intervals in each mouse. The first 2 samples of 75 µL each were taken from the retroorbital plexus under brief anesthesia and the third terminal sample was described for oral dosing. Plasma samples were obtained from whole blood by centrifugation at 2000 × *g* for 5 minutes at 4°C. Whole brain, liver, and intestine were excised and immediately frozen in liquid nitrogen. All samples were stored at −80°C until analysis.

### Analysis of Olanzapine and DMO

An ultra-high-performance liquid chromatography system consisting of Vanquish (Thermo Scientific) coupled with a high-resolution mass spectrometer with an orbitrap analyzer Exploris 120 (Thermo Scientific) was used in the quantitative analyses of olanzapine and desmethylolanzapine in the plasma and brain homogenates. Details of the analysis can be found in the Supplementary Materials.

### Rapid Equilibrium Dialysis

A RED device equipped with a single-use 48-well plate containing 8 kDa molecular weight cutoff (MWCO) inserts (Thermo Scientific Pierce) was utilized to determine the protein binding of olanzapine and DMO in the plasma and brain homogenates. Plasma samples collected from individual mice at each time point were pooled into composite samples due to insufficient sample volumes for performing RED. Similarly, brain homogenate samples from individual time points were pooled. RED was performed according to the manufacturer’s protocol (for details, see the Supplementary Materials). The free drug fraction in plasma was calculated by using Equation 1:


$$\begin{array}{l} f_{u,plasma}=\frac{c_{\left(buf\;fer\;chamber\right)}}{c_{\left(plasma\;chamber\right)}} \end{array}$$

The free drug fraction in the brain homogenate was determined by using Equation 2, which accounts for the effect of tissue dilution on the unbound fraction:


$$\begin{array}{l} f_{u,\;brain}=\frac{1/D}{\left(1/f_{u,diluted}-1\right)+1/D} \end{array}$$

were *D* is the dilution factor in the brain homogenate and the *f*_*u*,diluted_ associated free fraction determined as the ratio of concentrations in the buffer versus the diluted brain tissue.

### Pharmacokinetic Analysis

The extent of the systemic exposure to olanzapine and DMO was evaluated by using the AUC curves from 0 to the last time point of sampling (AUC_*t*_).^22^ The calculation of the point estimate and the 95% bootstrap *t*-confidence interval for AUC_*t*_ was performed with the help of the R-package PK (https://cran.r-project.org/web/packages/PK/index.html). Total brain-to-plasma drug partition coefficient (*K*_*p*,brain_) and unbound brain-to-unbound plasma partition coefficient (*K*_*p*,uu, brain_) were calculated using Equations 3 and 4:


$$\begin{array}{l} K_{p,\;brain}=\frac{AUC_{t,brain}}{AUC_{t,plasma}} \end{array}$$

$$\begin{array}{l} K_{p,\;\;\;uu,brain}=\frac{f_{u,brain}\;\times \;AUC_{t,brain}}{f_{u,\;\;\;plasma}\;\times \;AUC_{t,plasma}} \end{array}$$

**Figure 1.**
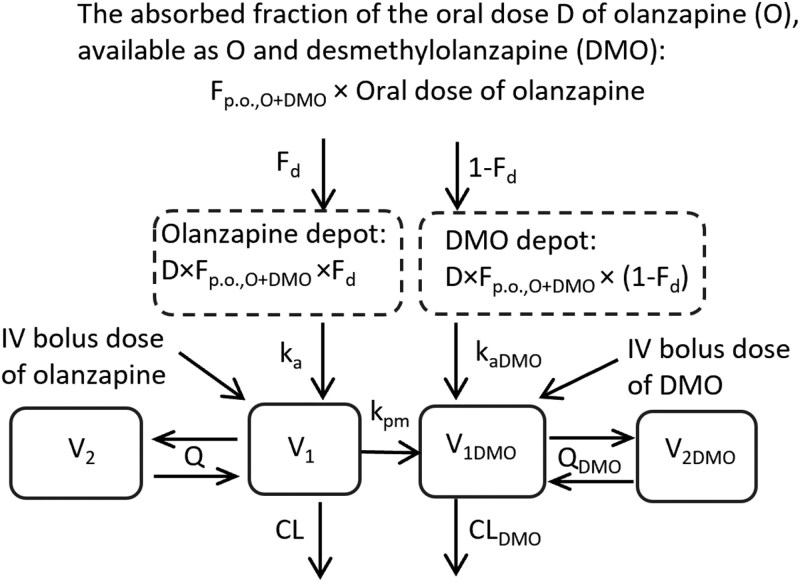
A schematic diagram of the olanzapine (O)–desmethylolanzapine (DMO) compartmental pharmacokinetic model. Here *V*_1_ and *V*_2_ represent the distribution volumes of central and peripheral compartments for olanzapine, and *V*_1DMO_ and *V*_1DMO_ are the distribution volumes of central and peripheral compartments for DMO. The intercompartmental clearances for olanzapine and DMO are denoted by the symbols *Q* and Q_DMO_ and plasma clearances by the symbols CL and CL_DMO_. Presystemic formation of DMO after oral (p.o.) olanzapine is modeled by splitting the absorbed fraction of the dose systemically available as either olanzapine or DMO (*F*_p.o., O+DMO_) into 2 parts, with the first part *F*_*d*_ absorbed as olanzapine at the absorption rate *k*_*a*_ in the central olanzapine compartment *V*_1_, and the other part (1 − *F*_*d*_) absorbed as DMO at the absorption rate *k*_*a*__DMO_ in the central metabolite compartment *V*_1DMO_. The absolute bioavailability of oral olanzapine (*F*_p.o._) is the product of (*F*_p.o.,O+DMO_ × *F*_*d*_). The formation of DMO from plasma olanzapine was accounted for by unidirectional mass transfer between the compartments *V*_1_ and *V*_1DMO_ at a rate described by a first-order conversion rate constant *k*_pm_. The partial metabolic clearance of olanzapine to DMO (CL_O→DMO_) is the product of (*V*_1_ × *k*_pm_).

Population compartmental modeling of olanzapine and DMO made use of the stochastic approximation expectation maximization algorithm combined with the Markov chain Monte Carlo procedure, as implemented in the Monolix software, version 2023.R1 (Lixoft). The individual values of the pharmacokinetic parameters were obtained as empirical Bayes estimates. The process of model building and validation is described in the Supplementary Materials. Definitions of all pharmacokinetic parameters and their acronyms are explained in the legend in Figure 1.

### Quantification of Gene Expression

Total RNA was isolated from the liver and brain tissue samples by using the TRIzol reagent (Invitrogen) and converted into cDNA via a high-capacity cDNA Reverse Transcription Kit (Applied Biosystems). Gene expression analysis was performed by reverse transcription polymerase chain reaction (qRT-PCR) with a QuantStudio 7 Flex Real-Time PCR System (Applied Biosystems). The amplifications were run by using the TaqMan Fast Universal PCR Master Mix and pre-designed TaqMan Gene Expression Assays (Table S1), which were all purchased from the Applied Biosystems Company. The amount of 30 ng cDNA was loaded into each reaction, which was all performed in duplicate. The time–temperature profile used in the “fast” mode was 95°C for 3 minutes; 45 times: 95°C for 7 seconds, and 60°C for 25 seconds. The target gene expression was calculated according to the 2^−ΔΔCt^ method. Gapdh (glyceraldehyde 3-phosphate dehydrogenase) or 18S rRNA genes were used as a reference for normalizing the data.

### Statistical Analysis

Statistical analysis was performed by using GraphPad Prism, version 5.0 (GraphPad Software). The results measured at a single time point, the time-invariant pharmacokinetic parameters, or cumulative measures (AUC_*t*_) were compared between the LPS and control groups by using the *t*-test. Data on mRNA expression, plasma IL-6, and other biochemical tests were evaluated by 2-way repeated-measures analysis of variance (ANOVA) by using the group as a between-subjects factor and time as a repeated measure, followed by the Holm–Sidak post hoc test. If the conditions of variance homogeneity were strongly violated, the characteristics were log-transformed. For all statistical procedures, *P* values <.05 were taken as being significant.

## RESULTS

### Protein Binding of Olanzapine and DMO in the Plasma and Brain Homogenates

In the LPS and control mice, the mean range of the unbound fractions of olanzapine in the plasma was 40.1% (32.5–44.6) and 41.6% (30.2–45.9) (*P* = .75), respectively, and those of DMO was 8.3% (5.3–9.2) and 13.0% (10.0–15.0) (*P* < .02) ng/mL, respectively. The corresponding unbound fractions of olanzapine in the brain homogenate samples were 29.1% (20.5–37.5) and 27.4% (22.3–34.1) (*P* = .59), respectively, and those of DMO were 8.8% (6.4–10.3) and 8.7% (6.2–11.1) (*P* = .96), respectively. For the control samples, the unbound fraction of clozapine in brain homogenate ranged from 1.4% to 2.1%, while for trazodone it ranged from 7.8% to 11.8%.

### Plasma Concentrations of Olanzapine and DMO After Single PO and IV Doses of Olanzapine

**Figure 2.**
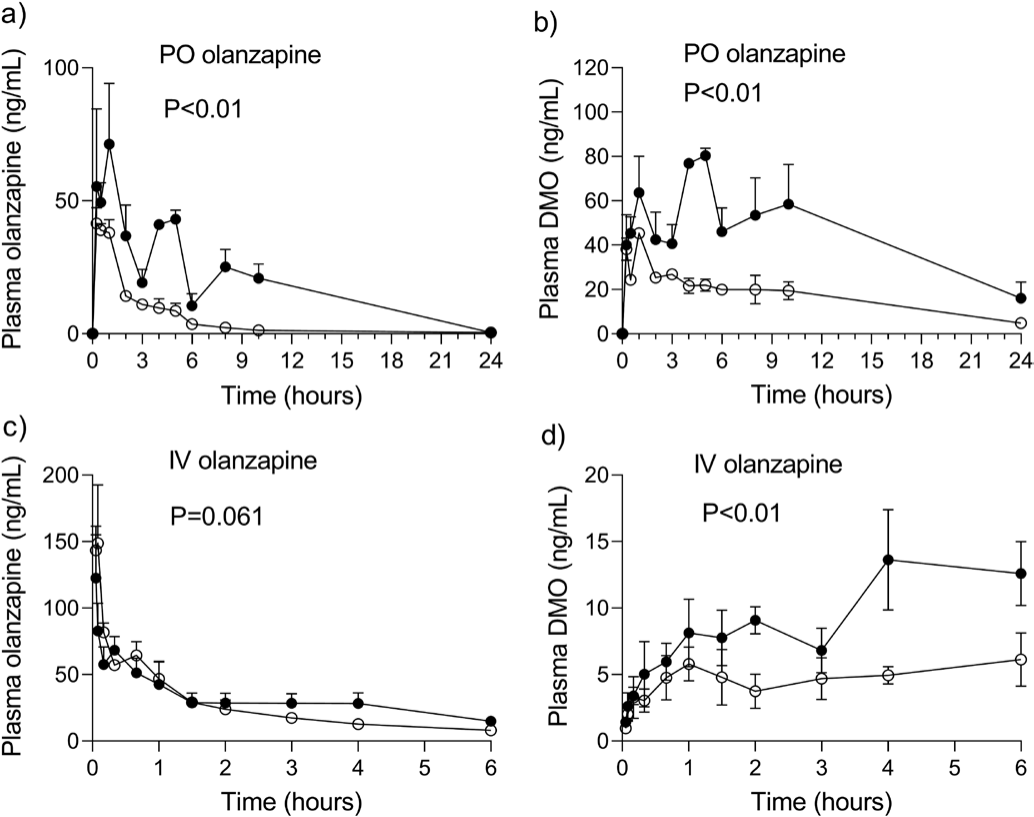
The mean (SEM) concentration–time curves for olanzapine and desmethylolanzapine (DMO) in the plasma of the mice injected with lipopolysaccharide (filled circles) and control mice (empty circles) after a single oral (PO) administration of 4 mg/kg of olanzapine (A, B) and, after a single intravenous (IV) bolus injection of 0.5 mg/kg of olanzapine (C, D). The *P*-value is given for the group effect from a 2-way repeated-measures ANOVA analysis of the concentration–time curves, with the group as a between-animal factor and time as a repeated measure.

After a single PO olanzapine dose of 4 mg/kg, the concentration–time profiles of olanzapine and DMO in the plasma were considerably higher in the LPS mice than in the controls (Figure 2A). In response to LPS, the mean AUC_24h_ of plasma olanzapine increased 3.8-fold and that of DMO 2.6-fold (*P* < .05) (Table 1). The effect of LPS on the plasma profile of olanzapine was noticeably less after an IV dose of 0.5 mg/kg olanzapine (Figure 2C) than after the PO dose. The AUC_6h_ of plasma olanzapine increased by 22% in the LPS mice relative to the controls (*P* < .05, Table 1), pointing toward a modest attenuation of the rate of drug elimination from the systemic circulation. Contrary to that, the concentrations of plasma DMO (Figure 2D) and the AUC_6h_ were 2-fold higher in the LPS mice than in the controls   (*P* < .05, Table 1). The DMO‐to‐olanzapine ratios of the mean AUCs (the metabolic ratios) in the LPS mice and the controls were 2.3 and 3.4 for PO olanzapine and 0.30 and 0.18 for IV olanzapine. Such differences in the DMO‐to‐olanzapine exposure ratios between the PO and IV routes of administration support the idea that the first-pass formation of DMO is significant.

**Table 1.** The AUC from 0 to the last time point of sampling (AUC_*t*_, hour × ng/mL) for olanzapine and DMO in the plasma of the LPS-challenged and control mice after a single oral (PO) administration of olanzapine at a dose of 4 mg/kg and, after a bolus intravenous (IV) injection of 0.5 mg/kg olanzapine or DMO.

		AUC_*t*_ (hour × ng/mL)^a^	
Dose	Compound	LPS	Controls
4 mg/kg PO olanzapine	Olanzapine	462 (339–585)*	121 (107–135)
	DMO	1070 (697–1440)*	408 (317–500)
0.5 mg/kg IV olanzapine	Olanzapine	192 (172–211)*	157 (143–171)
	DMO	57.6 (50.8–64.4)*	28.7 (23.8–33.6)
0.5 mg/kg IV DMO	DMO	1690 (1390–1990)	562 (521–604)

Abbreviations: AUC, area under the concentration–time curves; DMO, desmethylolanzapine; LPS, lipopolysaccharide.

Data are means (95% confidence intervals for the mean).

^a^The last time point of sampling “*t*,” was 24 hours for oral administration and 6 hours for intravenous administration.

*P < .05.

### Plasma Concentrations of DMO After a Single IV Dose of DMO

**Figure 3.**
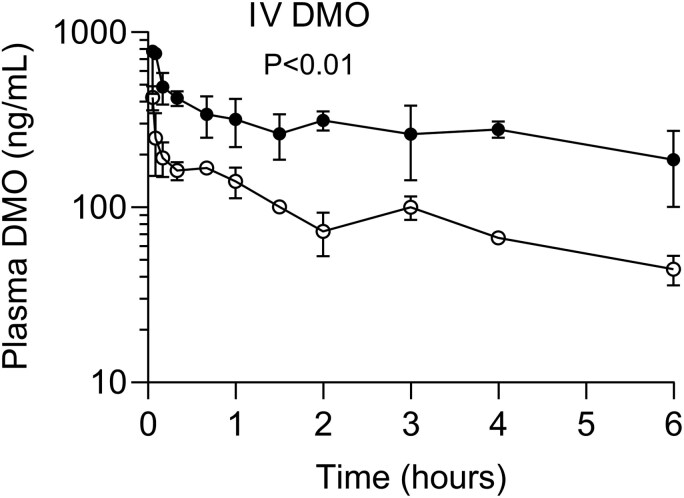
The mean (SEM) concentration–time curves for plasma desmethylolanzapine (DMO) in the mice injected with lipopolysaccharide (filled circles) and control mice (empty circles) after a single intravenous (IV) bolus injection of 0.5 mg/kg of DMO. The *P*-value is given for the group effect from a 2-way repeated-measures ANOVA analysis of the concentration–time curves, with the group as a between-animal factor and time as a repeated measure.

After an IV bolus injection of 0.5 mg/kg of DMO, the concentration–time curve for plasma DMO of the LPS-injected mice had a flattened terminal part (Figure 3) and, the AUC_6h_ was 3-fold higher than that of the controls (*P* < .05) (Table 1), both providing direct evidence that LPS considerably decelerates the metabolite’s elimination. Olanzapine was undetectable in the plasma after the IV administration of DMO.

### The Brain Penetration of Olanzapine and DMO

**Figure 4.**
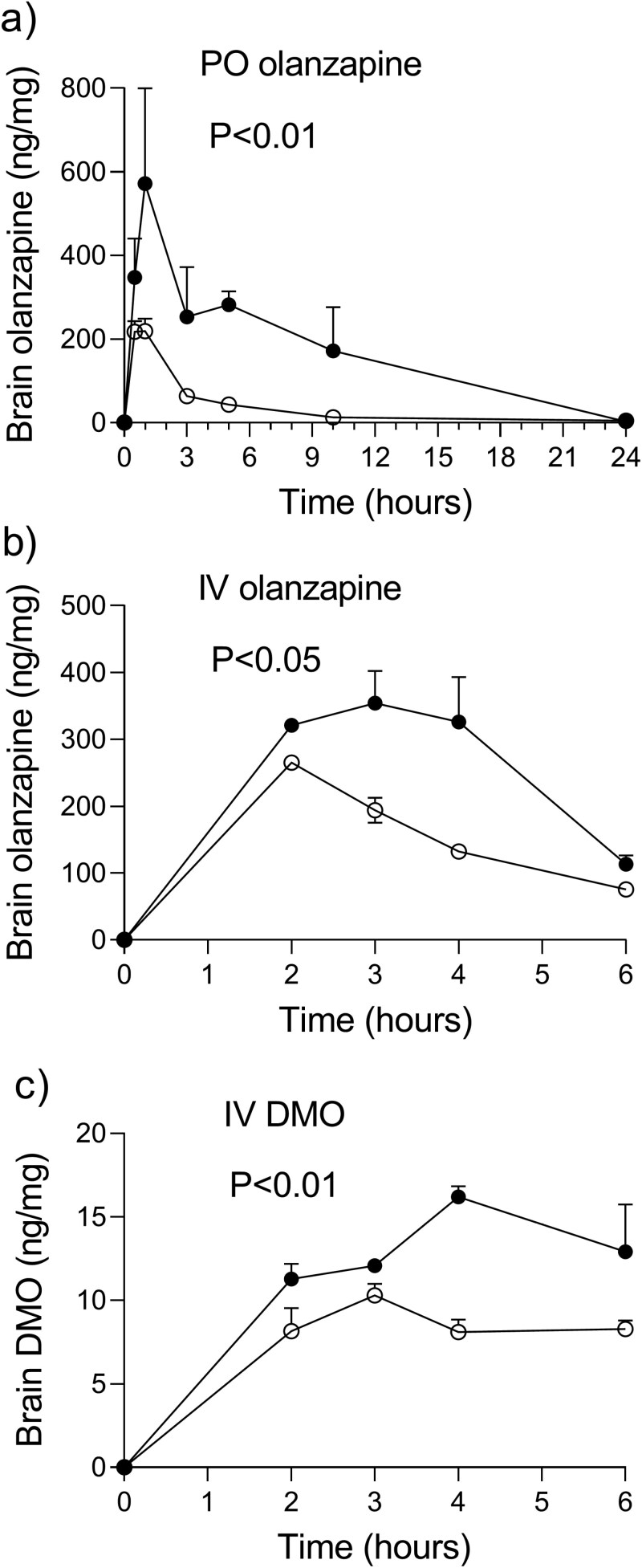
Mice injected with lipopolysaccharide (LPS) had higher concentrations of olanzapine and desmethylolanzapine (DMO) in the brain homogenate: Mean (SEM) concentration–time curves for olanzapine and DMO in the brain homogenates of the LPS mice (filled circles) and control mice (empty circles) after a single oral (PO) administration of 4 mg/kg of olanzapine (A), after a single intravenous (IV) bolus injection of 0.5 mg/kg of olanzapine (B) and, after a single IV bolus injection of 0.5 mg/kg of DMO (C). The *P*-value is given for the group effect from a 2-way repeated-measures ANOVA analysis of the concentration–time curves, with the group as a between-animal factor and time as a repeated measure.

Figures 4A and 3B show brain homogenate concentrations of olanzapine after single PO and IV administrations. In comparison to the controls, the LPS mice orally and intravenously dosed with olanzapine had the values of olanzapine AUC_24h_ in the brain 5.2-fold (*P* < .05) and 1.8-fold higher (*P* < .05) (Table 2). The brain-to-plasma ratios of the total concentrations (*K*_*p*,brain_) of 8.5 and 6.3, as well as those of the unbound concentrations (*K*_*p*,uu,brain_) of 6.2 and 4.1, both confirmed an augmented passage of olanzapine into the brain of the LPS animals as compared with controls. The brain penetration ratios *K*_*p*,brain_ and *K*_*p*,uu,brain_, obtained by using the brain-to-plasma ratios of olanzapine AUC_6h_ after an IV injection of olanzapine, were similar to those after PO dosing (Table 2).

**Table 2.** The brain penetration of olanzapine and DMO—the AUC from 0 to the last time point of the sampling (AUC_*t*_) for olanzapine (OL) and desmethylolanzapine (DMO) in the brain homogenates of the LPS-challenged and control mice after a single oral (PO) administration of olanzapine at a dose of 4 mg/kg PO and, after a bolus intravenous (IV) injection of 0.5 mg/kg of olanzapine or DMO.

	Assayed compound	AUC_*t*_ (hour × ng/mg)^a^		*K*_*p*,brain_		*K*_*p*,uu,brain_	
Dose		LPS mice	Controls	LPS mice	Controls	LPS mice	Controls
4 mg/kg PO OL	OL	3950 (1850–6040)*	758 (661–854)	8.5	6.3	6.2	4.1
	DMO	trace levels^b^	trace levels^b^	na	na	na	na
0.5 mg/kg IV OL	OL	1595 (1060–2240)*	866 (803–934)	8.3	5.5	6.0	3.6
	DMO	nd	nd	na	na	na	na
0.5 mg/kg IV DMO	OL	66.7 (53.9–77.8)*	43.0 (37.0–50.9)	0.039	0.077	0.042	0.051

Abbreviations: AUC, area under the concentration–time curves; DMO, desmethylolanzapine; LPS, lipopolysaccharide; na, not applicable; nd, not detected.

The data are mean values (95% confidence intervals for the mean).

^a^The last time point of sampling *t*, was 24 hours for the oral administration and 6 hours for intravenous administration.

^b^See the text for the results.

*P < .05.

The concentrations of DMO in the brain homogenates were below the detection limit of 0.20 ng/g in 4 of 18 and 5 of the 18 animals of the LPS and control groups dosed with PO olanzapine, with the highest assayed levels not exceeding 1.6 and 1 ng/mg. After IV olanzapine, neither the LPS animals nor the controls had any brain homogenate levels of DMO higher than the limits of detection. Contrary to that, the concentrations of DMO in the brain homogenates were higher than the limits for quantification at the termination intervals in all the mice IV injected with DMO (Figure 4C). The *K*_*p*,brain_ values of 0.039 and 0.077 and the *K*_*p*,uu,brain_ of 0.042 and 0.051, in the LPS and control mice, indicate that the brain penetration of DMO is minimal (Table 2).

**Figure 5.**
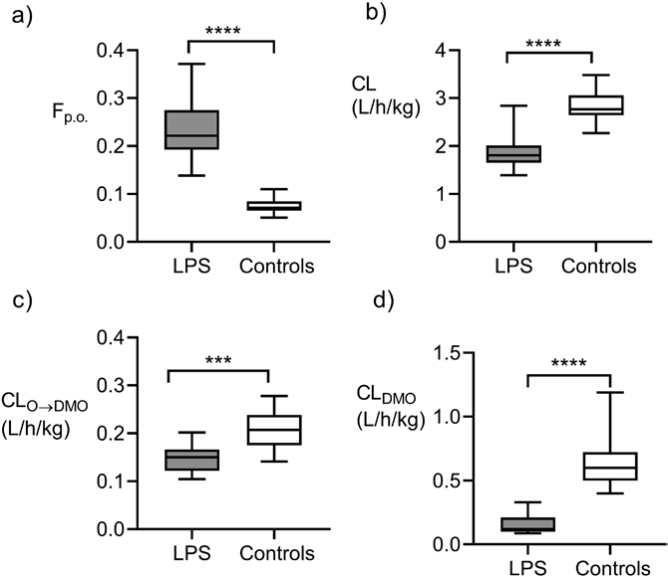
Lipopolysaccharide-induced changes in the pharmacokinetic characteristics which influence the systemic exposure to olanzapine and desmethylolanzapine (DMO): (A) absolute bioavailability of oral olanzapine (*F*_p.o._), (B) plasma clearance of olanzapine (CL), (C) partial metabolic clearance of olanzapine to DMO (CL_O→DMO_), and (D) plasma clearance of DMO (CL_DMO_). The box plots show the medians, interquartile ranges, and ranges. ****P < .0001, ***P < .001, according to the *t*-test.

The pharmacokinetic modeling of olanzapine and DMO was aimed at achieving a more detailed evaluation of the LPS-induced changes in the pharmacokinetics of both compounds. The best compartmental pharmacokinetic model is depicted in Figure 1, and definitions of all pharmacokinetic parameters and their acronyms are explained in the legend. The most profound change in the exposure-related pharmacokinetic characteristics of olanzapine, induced by LPS, was an increased absolute bioavailability *F*_p.o._ from 0.075 to 0.235 (a 3.1-fold difference). Contrary to that, the plasma clearance of olanzapine (CL) showed only a modest decrease from the median control value of 2.84 to 1.85 L/h/kg in the LPS-injected mice, that is, by 35% (Figure 5 A, B). The LPS attenuated the plasma clearance of DMO (CL_DMO_) by 79% (Figure 5D).

Thus, a noticeable difference in the pharmacokinetics between DMO and olanzapine was an almost 5-fold lower CL of the former and a higher extent of its attenuation by LPS. According to the joint parent–metabolite pharmacokinetic model, the partial metabolic clearance of plasma olanzapine to DMO (CL_O→DMO_) was less in the LPS mice than in the controls (median values of 0.135 and 0.26 L/h/kg, *P* < .001, Figure 5C). Moreover, the median fraction of the absorbed amount of olanzapine presystemically converted to DMO (1-Fd) was 0.14 (range: 0.10–0.15) and 0.26 (range: 0.24–0.28) in the LPS mice and the controls (*P* < .001), supporting the claim that LPS reduces the first-pass metabolism of olanzapine to DMO. However, because of a higher absorbed fraction of the olanzapine dose in the former group, the absolute bioavailability of DMO was comparable in both groups: 0.030 (the range: 0.014–0.037) and 0.029 (0.025–0.035) (*P* = .080). Supplementary Materials contain details of modeling, graphical and tabular outputs, and the statistical evaluation of the pharmacokinetic models.

### Changes in the mRNA Expression of the Inflammatory Mediators, Enzymes, and Transporters

**Figure 6.**
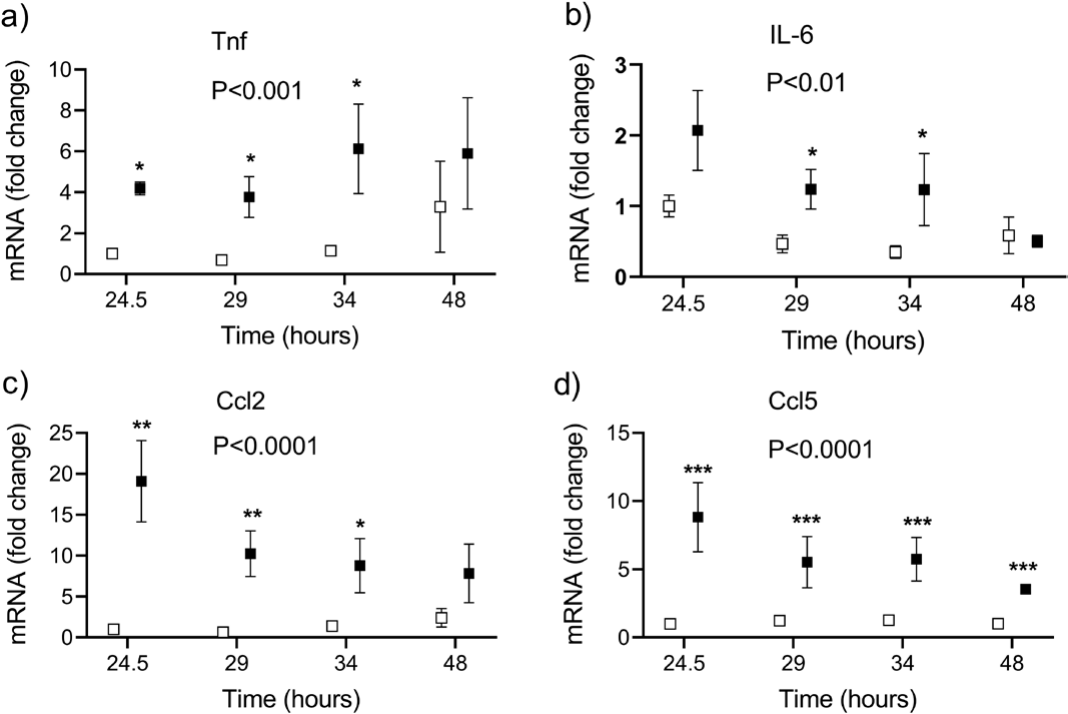
The effect of the lipopolysaccharide (LPS, 4 mg/kg IP at the time 0 hours) on the mRNA expression of the inflammatory cytokines and chemokines tumor necrosis factor (TNF), interleukin-6 (IL-6), C–C motif chemokine ligand 2 (CCL2), and C–C motif chemokine ligand 5 (CCL5), in the mouse livers. Statistical significance was determined by using a 2-way ANOVA with a Holm–Sidak’s post hoc test after a logarithmic transformation. A *P*-value for the overall group effect is given (*N* = 18/group). Asterisks denote the statistical differences between the LPS-injected (black squares) and control (white squares) mice at particular time intervals: *P < .05, **P < .01, ***P < .001.

**Figure 7.**
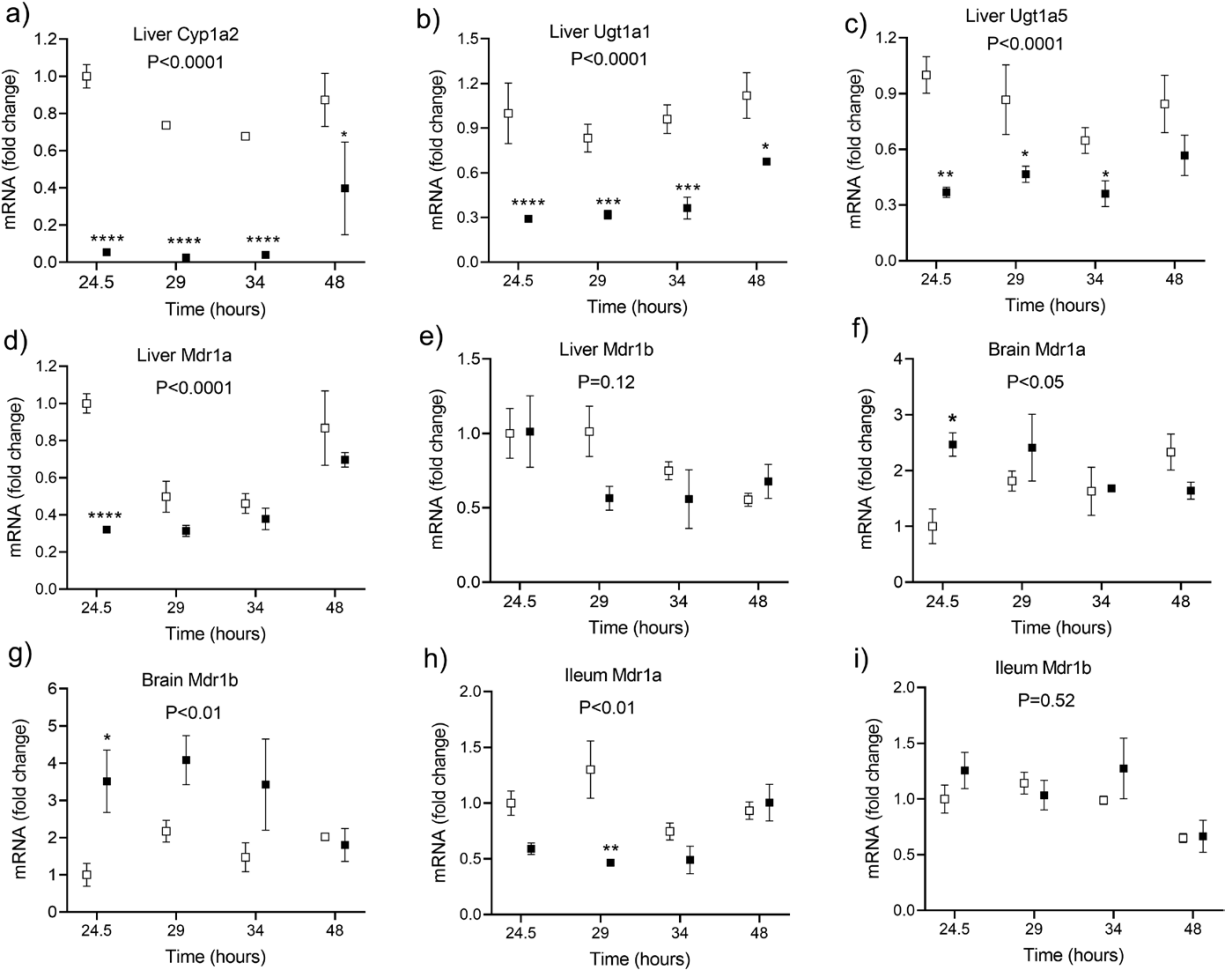
The effect of the lipopolysaccharide (LPS, 4 mg/kg IP at the time 0 hour) on the mRNA expression of the enzymes cytochrome P450 1A2 (Cyp1A2), UDP glucuronosyltransferase 1A1 (Ugt1A1), and UDP glucuronosyltransferase 1A5 (Ugt1A5), in the mouse livers, and of membrane transporters multidrug resistance proteins 1a (Mdr1a) and 1b (Mdr1b), in the mouse livers, brain, and ileum. The statistical significance was determined by using a 2-way ANOVA with a Holm–Sidak’s post hoc test after a logarithmic transformation. A P-value for the overall group effect is given (*N* = 18/group). Asterisks denote statistical differences between the LPS-injected (black squares) and control (white squares) mice at particular time intervals: *P < .05, **P < .01, ***P < .001.

In the livers harvested between 24 and 48 hours following LPS injection, the mRNA expression was induced of genes encoding inflammatory cytokines and chemokines TNF, IL-6, CCL2 (monocyte chemoattractant protein 1), and CCL5 (RANTES) (Figure 6). A significant downregulation of the mRNA expression of Abcb1a (P-glycoprotein) in the liver and ileum was detected, whereas Abcb1b was comparable to the controls. The upregulation was observed in the Abcb1a and Abcb1b mRNAs in the brain. Among the tested enzymes in the liver, the most important attenuation by LPS was found for the mRNA of Cyp1A2, which decreased by more than 90%, followed by the UGT enzymes with their mRNA expression at around 30% of the saline-injected controls (Figure 7).

### Verification of the Murine Endotoxemia Model

Within 24 hours of the IP injection, the survival rate was 100% in the groups of 32 animals treated with 4 mg/kg LPS and 30 saline-treated animals. Two mice from the LPS group injected with olanzapine died after the first withdrawal of blood and were replaced. At 24 hours, the control animals showed an average weight loss of 2.1% body weight, while the LPS-injected mice lost an average of 12.8 % body weight (*P* < .001). Table S4 summarizes the results of the biochemistry tests. The albumin-to-globulin ratio in the plasma of the LPS group of 1.5 ± 0.42 was less than the ratio of 2.9 ± 0.43, which was detected in the controls (*P* < .001). Alpha1-acid glycoprotein, the acute-phase protein involved in olanzapine protein binding in the plasma, was induced to the concentration of 407 ± 136 mg/L (*P* < .001) by LPS injection, well above the control value of 157 ± 21 mg/L in the saline-treated mice. The median (interquartile range) plasma concentrations of IL-6 of 98 (47-212) in the LPS-injected mice were markedly higher than the results in controls of 10.7 (9.7-14) (*P* < .001).

## DISCUSSION

A principal finding of the present study is that systemic and CNS exposure to olanzapine is considerably increased after a single PO administration of the drug to endotoxemia-induced C57BL/6N mice as compared to the control mice not injected with LPS. The LPS-induced increase in the AUC_24h_ of plasma olanzapine can be ascribed, mostly to the 3-fold augmented absolute bioavailability of the drug, and to a small extent, its modestly attenuated systemic CL. The higher values for the *K*_*p*,brain_ and *K*_*p*,uu,brain_ of olanzapine, indicate the augmented passage of olanzapine into the brain of the LPS-injected mice, which significantly contributes to the 5-fold higher exposure of the target organ to olanzapine, besides the 3.8-fold increased concentrations of plasma olanzapine. Another result that deserves more attention is the minimal brain penetration of DMO in comparison with olanzapine in both the LPS-injected and control mice.

The CL/F value for PO olanzapine in the control mice of 33.1 L/h/kg is higher than the result of 9.9 L /h/kg previously observed after an PO dose of 15 mg/kg,^21^ and similar to the value of 27.7 L/h/kg in mice dosed with 10 mg/kg of olanzapine.^23^ Comparatively lower values for the CL/F of 1.7 and 2.4 L/h/kg have been reported after IP injections of the mice with 2 and 25 mg/kg of olanzapine.^24,25^ A major difference in the CL/F after PO versus IP administration confirms the existence of an extensive first-pass effect, supporting our direct measurement of low absolute bioavailability of PO olanzapine in the mice. The plasma CL of 2.8 L/h/kg for olanzapine in the control mice approximately equals the average plasma flow through the murine liver, indicating a high hepatic extraction ratio. The attenuation of the hepatic enzymes has a lower relative effect on the plasma CL of drugs efficiently extracted by the liver. Contrary to that, the relative reduction of the first-pass elimination of such drugs is larger, as is the increase in the *F*_p.o_ value. In line with this theory is the modest attenuation of the systemic CL by only 35 % as well as the disproportionally larger, several-fold increases in the *F*_p.o._ and AUC_24h_ of olanzapine in the LPS mice. The downregulation of P-gp in the intestine, accompanied with increased absorption, might have contributed to the LPS-induced increase in the AUC_24h_ of PO olanzapine, besides the reduced first-pass metabolism. The plasma CL of the highly extracted drugs is sensitive to reductions in the blood flow through the liver. However, the serum biochemistry showed no signs of ischemic injury to the liver and kidneys of the LPS mice due to their decreased cardiac output. Other investigators have shown that the left ventricular systolic pressure and cardiac output recover after 20 hours, from the IP injection of the male BALB/c mice with 5 mg of the *E. coli* LPS.^26^ The transient nature of the LPS-induced cardiovascular dysfunction thus excludes a large difference in the hepatic blood flow between the LPS and the control mice.

The ability to penetrate the blood–brain barrier is one of the most important factors influencing the efficacy of antipsychotics, together with the extent of their binding to the plasma proteins and their specific and nonspecific binding in the CNS.^27^ The concentration–time curves and the *K*_*p*,brain_ values for total olanzapine document the fast and excessive penetration of the drug into the murine brain. In concordance, the AUC_*t*_ of olanzapine was 8 times larger in the brain than in the plasma after a single oral dose of 6 mg/kg to the rats.^28^ A cutting-edge study on the regional distribution of antipsychotics in the rat brain found the *K*_*p*_ values for total olanzapine in the various brain regions between 1.9 (cerebellum) and 4.1 (frontal cortex) and the *K*_*p*,uu_ values between 0.86 (brainstem) and 1.8 (frontal cortex).^29^ The same authors have been able to document that olanzapine is unique among antipsychotics in its ability to accumulate in the lysosomes of the neural cells in a cationic form, reaching the intracellular-to-interstitial ratio of the unbound concentrations (*K*_*p*,uu,cell_) of 5.3 in the cortex and striatum. The *K*_*p*,uu, brain_ value higher than one, supports the view of the unrestricted passage of olanzapine across the blood–brain barrier, which can be further facilitated by the lysosomal accumulation of olanzapine cations. Therefore, the role is most likely limited to efflux transporters of the blood–brain barrier, including P-glycoproteins (P-gp). Olanzapine is transported by P-gp in the mice.^30^ However, its affinity for P-gp is relatively low, especially in comparison with clozapine and many other antipsychotics.^31,32^ Despite the upregulation of the mRNA expression for Abcb1a and Abcb1b in the brain, the *K*_*p*,uu,brain_ value for olanzapine in the LPS-injected mice was higher than in the controls. The results of the in vitro and in vivo studies support the idea that efflux from the brain can be decreased by the substrates for P-gp, due to the posttranscriptional inhibition of its activity, even when the expression of the transporter protein is increased by inflammation.^33-35^ The unbound fractions of olanzapine in the plasma and brain homogenates were similar in both groups. The concentrations of albumin and alpha1-acid glycoprotein, the 2 olanzapine-binding proteins in the plasma, showed opposite changes in response to endotoxemia. To confirm our results, the plasma protein binding of olanzapine was similar in healthy subjects and in patients with severe renal insufficiency, accompanied with hypoalbuminemia and an increased concentration of alpha1-acid glycoprotein.^36^ The large differences between the brain penetration of olanzapine and DMO argue against the disruption of the blood–brain barrier by LPS, accompanied by increased transcellular and paracellular passage of molecules. The brain penetration of the metabolite is less since DMO is less lipophilic and more bound to plasma proteins than olanzapine. Moreover, the pK_a_ of the protonated form of DMO is 8.8 and, the cation is incapable of passive diffusion into the lysosomes. Lysosomal ion trapping of DMO in the neural cells is thus hindered unlike that of olanzapine with a pK_a_ of 7.2.

In mice as well as in humans, olanzapine’s metabolic pathways include N-demethylation.^9,21^ The study by Uehara et al. and this study show that the concentrations of DMO in the murine plasma exceed those of olanzapine, unlike in humans.^23^ A markedly lower systemic CL of DMO in comparison to olanzapine CL in mice likely explains the observed shift in the metabolite-to-parent ratio of concentrations. Despite the lowering effects of LPS on the extent of first-pass metabolism, the plasma clearance, and the partial metabolic clearance of olanzapine to DMO, the DMO concentrations were higher in the plasma of the endotoxemia mice, primarily due to the markedly reduced metabolite CL, in comparison to the controls. The DMO-to-olanzapine metabolic ratio of concentrations is considered to be a good predictor of the plasma CL and the steady-state concentration-to-dose ratios of olanzapine in the patients.^1,9^ This concept might not be universally applicable, since the metabolism of olanzapine was inhibited, and the metabolic ratio increased due to a disproportionally larger inhibitory effect of LPS on the elimination of DMO. There exist similarities as well as major differences between mice and humans in the expression and activities of the CYPs and UGT enzymes. The UGT1A4-catalyzed glucuronidation of olanzapine is unique for humans. The mRNA expression of the UGT 1A1 and 1A5 enzymes was reduced in the liver of the LPS-injected mice, in parallel with CYP1A2. Physiologically based models of olanzapine pharmacokinetics in humans show that UGT1A4- and CYP1A2-mediated metabolic clearances are important determinants for the AUC of oral olanzapine.^37^ In agreement with our findings in the endotoxemia mice, the catalytic activities of several CYPs, including CYP1A2, and the activity of UGT1A were shown to decrease within 24–48 hours of exposure of the primary human hepatocytes to the pro-inflammatory cytokines TNFa, IL-6, and IL-1beta.^38^ Thus, the results of the present study might predict, at least to some extent, the changes in the pharmacokinetics of oral olanzapine in acutely infected patients. Considering a lower hepatic extraction ratio of 0.3 for olanzapine in humans as compared to mice,^9^ the inflammation-induced changes in CYP1A2- and UGT1A4-mediated clearances can be expected to substantially impact both the AUC and the half-life of olanzapine in the patients.

LPS is the primary endotoxin involved in inflammatory processes, sepsis, and multiorgan failure caused by Gram-negative bacteria like *E. coli.*^39^ The biological effects of a moderate non-lethal dose of LPS (5 mg/kg) to C57BL/6 mice have been repeatedly described elsewhere, including the suppression of hepatic enzyme activities of the CYP450 enzymes which is maximal between 12 and 24 hours and slowly recovers until 72-hour postinjection.^40-43^ The concentrations of IL-6 in the plasma and the hepatic mRNA expression of the inflammatory cytokines and chemokines proved their robust induction by LPS. It is one of the study imperfections that the investigation was limited to the mRNA expression of the CYP1A and UGT enzymes involved in the 2 most important pathways of olanzapine metabolism. Due to the inherent limitations of the murine model of sepsis, the extrapolation of the results should be done with caution in acutely infected patients treated with olanzapine. The rapid activation of the murine innate immune system by LPS produces a substantial but transient increase in the inflammatory cytokines which differs from that observed in the infected patients. The IL-6 concentrations in the LPS mice reported here are within the range of the cutoff values for serum IL-6 which is used to diagnose human sepsis.^44,45^

Results of animal and human studies support the view that intestinal microbiota has an important role in olanzapine-induced metabolic dysfunction.^46^ Repeated oral dosing of olanzapine to rodents alters the gut microbiota to obesogenic and pro-inflammatory profile causally related to metabolic side effects of the drug.^47^ Suppression of the rodent microbiota using antibiotics attenuates olanzapine-associated metabolic dysfunction.^48^ Interestingly, the bioavailability of oral olanzapine was increased 1.8-fold in rats that had undergone depletion of gut microbiota by three antibiotics simultaneously administered for 14 days. The effect was ascribed to the reduced first-pass glucuronidation of olanzapine in the intestine.^49^ A single administration of DMSO and MC as excipients unlikely had different effects on olanzapine pharmacokinetics in the LPS and control groups (an in-depth discussion is available in the Supplementary Materials). However, long-term administration of olanzapine admixed in a diet would better mimic oral pharmacotherapy of patients, enabling to evaluate the mutual interaction between the intestinal microbiota and olanzapine during sepsis.

In conclusion, the results of the first comprehensive investigation of olanzapine pharmacokinetics in endotoxemia mice clearly indicate that acute inflammations accompanying infections can considerably increase the systemic and brain concentrations of olanzapine after oral administration. Further studies should clarify whether the inflammation-induced changes in the pharmacokinetics of oral olanzapine result in brain overexposure to the drug and adverse effects in acutely infected patients.

## Supplementary Material

pyaf036_suppl_Supplementary_Tables_S1-S4

## Data Availability

The authors confirm that the data supporting the findings of this study are available within the article and its Supplementary Materials. Individual values are available upon request.
