# Supplementary material for: Acute inflammation induced by the Escherichia coli lipopolysaccharide considerably increases the systemic and brain exposure of olanzapine after oral administration in mice
Source: Int J Neuropsychopharmacol. 2025 May 24;28(6):pyaf036. doi: 10.1093/ijnp/pyaf036 (PMC12202309; doi:10.1093/ijnp/pyaf036)
Supplement: pyaf036_suppl_Supplementary_Tables_S1-S4 [file pyaf036_suppl_supplementary_tables_s1-s4.docx]

**Drug Administration. Doses and Biological effects of Excipients.** Olanzapine is a Biopharmaceutical Classification System Class II drug which shows low solubility in water but high permeability through biological membranes. Gastric gavage was used to ensure precise and accurate dosing. The drug substance was first dissolved in dimethyl sulfoxide (DMSO) and then quickly mixed with an aqueous solution of methyl cellulose (MC) as an excipient. The gavage volume of 5 mL/kg (0.5% body weight) is considered appropriate for rodents.^1^ The volume load to mice weighing 25 g was 125 microliters. The doses of DMSO and MC were 276 mg/kg and 25 mg/kg. The water content in the gastrointestinal tract in the fed and fasted mouse is 0.98 ± 0.4 and 0.81± 1.3 mL and, the stomach capacity is approximately 0.4 mL. Owing to dilution of DMSO in luminal fluids and its rapid absorption and distribution, the luminal concentration of the solvent was most probably less than 1%.^2,3^

DMSO is a polar aprotic solvent, miscible with water, which is frequently used for *in vitro* and *in vivo* procedures, including solubilization of poorly soluble drugs in permeation assays. The FDA classified DMSO as a class 3 solvent, which is the safest category. The acute toxicity of DMSO is low, the LD50 in mice is 16.5-24.6 g/kg p.o.^4^ No toxicity was observed (clinical observations, body weight, urinalysis, haematology, blood biochemistry, necropsy, organ weights, and histopathology) in male Sprague Dawley rats dosed over 5 days by oral gavage with 500 mg/kg DMSO at the concentration of 10 % (w:v) in a volume of 5 mL/kg.^5^ In cellular and tissue-based systems used to predict the absorption of drugs, DMSO at the final concentrations between 1% and 5% did not disturb the membrane integrity and, exerted no influence on the paracellular permeation of compounds. Under same conditions, DMSO showed a modest effect, if any, as an enhancer of transcellular permeation of poorly water-soluble drugs.^6,7^ In another study, the effects of DMSO and other solubilizing agents on intestinal epithelium barrier function and intestinal membrane damage were assessed in Male Wistar albino rats using an in vitro diffusion chamber and an in situ closed loop technique. DMSO (10 %, v:v) did not alter the in vitro paracellular transport of carboxyfluorescein (a soluble, membrane-impermeant, paracellular marker), across the intestinal sheets prepared from jejunal and ileal segments. In closed jejunal loop experiments, 10 % DMSO in a total volume of 1 mL had no effect on the absorption of carboxyfluorescein and did not augment the release of protein and lactate dehydrogenase from the intestinal membrane.^8^ An in vivo rat study evaluated the effect of 15 % (w:v) DMSO (660 mg/kg) in 0.5 % MC, dosed p.o. in a volume of 4 mL/kg, on the concentration-time profiles for cilostazole (a BCS Class II drug) in the plasma, stomach and the middle and lower segments of the small intestine.^9^ DMSO was rapidly absorbed and detected only in the stomach and duodenum, but not in the middle small intestine. DMSO increased the concentrations of dissolved cilostazole in the stomach and duodenum and the AUC for plasma cilostazole by 32%. In murine models in vitro and in vivo, DMSO attenuates LPS-induced cytokine production and inflammatory response.^10,11^ However, LPS was injected 24 hours before olanzapine administration. The data of the present study document the immune system response to LPS. Organic solvents inhibit the enzyme activities in a species-, concentration-, isoform-and substrate-dependent manner. Phenotyping of metabolic enzyme activities showed that the inhibitory effect of DMSO at a concentration of 1% is minor for rat enzymes involved in the metabolism of olanzapine (CYP1A, CYP2D, FMO) and for human UGTs. ^12-14^ Cellulose ethers, including methylcellulose (MC), are among the least toxic excipients, commonly used in drug formulations and for oral administration of drugs to animals. They are well tolerated and easy to prepare and handle. MC is commonly used in food as a thickener, emulsifier and stabilizer. The dose volume of 10 mL/kg of MC administered over 5 days by oral gavage in male Sprague Dawley rats induced no toxicity at the concentration of 0.25 % (w:v), whereas the concentration of 0.5 % modestly increased urine calcium levels without altering serum calcium.^5^ Cellulose derivatives form hydrogels that are biodegradable and biocompatible. MC is virtually unabsorbed in the GIT and fermented in the colon by microbiota. Hydroxypropyl methylcellulose (0.2 %), a nonionic cellulose derivative similar to MC, increased the intestinal absorption of atenolol during an in situ intestinal perfusion in Wistar rats. However, atenolol is a BCS Class III drug with a low permeability, considered to be much more susceptible to the effects of excipients as compared to the Class II drugs like olanzapine.^15^ Chassaing et al. reported that chronic exposure of C57BL/6  mice to carboxymehyl cellulose (1 % w:v) in drinking water over 12 weeks altered intestinal microbiota, induced low-grade inflammation and obesity/metabolic syndrome and increased gut permeability which correlated with increased levels of serum antibodies to flagellin and LPS. ^16^

In conclusion, the data from rodent studies support the view that a single administration of DMSO and MC at the doses used in the study is well tolerated, does not damage the intestinal epithelia, does not disturb the intestinal membrane integrity and, does not increase paracellular penetration of substances. The mixture of DMSO and MC increases the soluble fraction of lipophilic drugs in the intestine, accelerating the absorption. A modest, transcellular permeation-enhancing effect, observed in the drugs with a low permeation, may not be important for olanzapine, a BCS class II drug. A single administration of dimethyl sulfoxide and methyl cellulose as excipients unlikely had different effects on olanzapine pharmacokinetic in the lipopolysaccharide and control groups.

**Analysis of Olanzapine and DMO.** An ultrahigh-performance liquid chromatography system consisting of Vanquish (Thermo Scientific, San Jose, CA, USA) coupled with a high-resolution mass spectrometer with an orbitrap analyser Exploris 120 (Thermo Scientific, San Jose, CA, USA) was used in the quantitative analyses of olanzapine and desmethylolanzapine in the plasma and brain homogenates. The method was validated according to the bioanalytical method validation guidelines. ^17,18^ All of the evaluated parameters met the required criteria. Prior to analysis, 20 μL of plasma or brain homogenates were precipitated with 80 μL of ACN with an internal standard. The mixture was vortexed for 1 min at 1500 rpm and centrifuged at 12,000 g for 5 min at 20 °C. The supernatant was transferred to an injection vial and analysed. The chromatographic separation was carried out on a Luna Omega 1.6 μm Polar C18, 50 x 2.1mm ID (Phenomenex, Torrance, CA, USA), which is protected with a guard column Security Guard Ultra C18 (Phenomenex, Torrance, CA, USA). The separation with a total run time 4.5 minutes was performed in a gradient elution mode with a flow rate of 0.6 mL/min with the following composition of the mobile phase: A) 0.1% FA in water (v/v) and B) 0.1% FA in ACN/MeOH at a ratio of 50/50 (v/v). The gradient program was as follows: 0% of solvent B in 0–0.33 min, 0–100% of solvent B in 0.33–2.33 min, 100% of solvent B in 2.33–3.42 min and column equilibration with 0% of solvent B in 3.42–4.5 min. The column was kept at 45 °C during analysis. The samples were kept at 10 °C in a lighttight autosampler unit. A Heated-Electrospray Ionization II interface (HESI-II) in a positive ion mode was used with the following settings: spray voltage at +3.5 kV, S-lens RF level at +70 V, capillary temperature at 350 °C, auxiliary gas heater temperature at 350 °C, sheet and auxiliary gas flow at 50 and 15 arbitrary units, respectively. Full MS mode was used at a resolution (m/𝛥m) of ̴35,000 for the analysis of olanzapine ([M+H]^+^ = 313.14814), desmethylolanzapine ([M+H]^+^ = 299.13249) and internal standard olanzapine-*d*8 ([M+H]^+^ = 321.19836). Thermo Xcalibur software (v 4.0) was used for data evaluation.

**Rapid Equilibrium Dialysis.** Briefly, 50 µL of drug-spiked plasma or 100 µL of 10-fold diluted brain homogenates were added to the sample chambers of the RED device, while 300 µL or 350 µL of phosphate-buffered saline (PBS, pH at 7.4) were added to the corresponding buffer chambers. Each pooled sample was analysed in triplicate. Control samples consisting of plasma and brain homogenates from the study, spiked with clozapine and trazodone at a target concentration of 100 ng/mL, were analysed in each measurement series. Each plate was sealed with aluminium foil and incubated at 37 ± 1 °C with an agitation at 350 rpm on an orbital shaker (Randox PHMP, Grant Instruments Ltd, Shepreth, Cambridgeshire, England) for 4 hours. After incubation, a matrix-matching procedure was performed: 10 µL of each post-dialysis sample was mixed with an equal volume of PBS, while 10 µL of each buffer sample was mixed with the corresponding blank plasma or brain homogenate. Prior to analysis, 20 µL of the mixed plasma or brain homogenate samples were precipitated with 80 µL of acetonitrile containing an internal standard.

**Pharmacokinetic modelling of olanzapine and DMO**. First, the concentrations of olanzapine in the plasma after PO and IV administrations of the drug were simultaneously modelled. In the second step, the concentration-time data after the intravenous administration of DMO were modelled to assess distribution and elimination of the metabolite. Finally, simultaneous modelling of olanzapine and DMO was conducted after the conversion of the concentrations of DMO to olanzapine equivalents. The PK parameters of DMO were fixed to the values estimated after a single IV dose of the metabolite. Presystemic formation of DMO from orally administered olanzapine was modelled by splitting the absorbed fraction of the dose F_p.o._ (olanzapine+DMO) into two parts, with the first part F_d_ , absorbed as olanzapine at the rate k_a_ , in the central olanzapine compartment V_1_ and the other part, F_DMO_ = (1-F_d_) absorbed as DMO at the rate k_aDMO_ , in the central metabolite compartment V_1DMO_. To account for the metabolism of plasma olanzapine to DMO, a unidirectional transfer was assumed between the compartments V_1_ and V_1DMO,_ at a rate described by a first-order conversion rate constant k_pm_. The goodness-of-fit achieved by the pharmacokinetic models was assessed with the help of the objective function value and diagnostic plots of 1/observed and predicted concentrations vs. time, 2/ observed vs. individually predicted concentrations, 3/ weighted residuals vs. time and 4/ weighted residuals vs. predicted concentrations. Finally, the mean prediction errors and the mean absolute prediction errors were calculated.

The best disposition model for oral olanzapine was an open two-compartment model with first-order absorption and elimination. The between-animal variability in model parameters were described by an exponential model and a proportional model was used to describe the residual variability. The estimates for pharmacokinetic parameters of the model are listed in the Tables S2 and S3. Inclusion of the group (LPS mice, Controls) to the model as a binary categorical covariate for F_p.o._ and CL resulted in the decreases in the objective function value by 23 and 6.8 (P < 0.01), proving the significant effect of LPS on both pharmacokinetic parameters. The goodness of fit plots confirmed adequate prediction of individual concentrations by the model (Figures S1-S4). Imprecise estimates were obtained for some parameters, likely because of the small number of animals and sampling intervals on the ascending part of the concentration-time curves. Altogether, individually predicted concentrations agreed well with the assayed ones: the mean prediction error was -1.3 % and the range for 95% of differences between the predicted and observed concentration was from -23 to 20 %. Similar to olanzapine, the best disposition model for DMO after i.v. injection of DMO was an open two-compartment model with first-order elimination. Injection with LPS had significant effects on the CL and distribution volumes V_1_ and V_2_ (Table S2). In comparison to controls, their median values were decreased by 78.5 %, 70% and 43%. The goodness of fit plots confirmed the predictive power of the model (Figures S5 and S6). The mean prediction error was only 0.83 % and the range for 95% of differences between the predicted and observed concentrations was in the narrow range from -18 to 19%.

**Table S1.** TaqMan® Gene Expression Assays (Applied Biosystems) used for qRT-PCR.

| Gene symbol | Name | Life Technologies Assay ID |
| --- | --- | --- |
| 18S | 18S ribosomal RNA | Hs99999901_s1 |
| Abcb1a | ATP-binding cassette, sub-family B member 1A (Mdr1a) | Mm00440761_m1 |
| Abcb1b | ATP-binding cassette, sub-family B member 1B (Mdr1b) | Mm00440736_m1 |
| Ccl2 | C-C motif chemokine ligand 2 | Mm00441242_m1 |
| Ccl5 | C-C motif chemokine ligand 5 | Mm01302427_m1 |
| Cyp1a2 | cytochrome P450, family 1, subfamily a, polypeptide 2 | Mm00487224_m1 |
| Gapdh | glyceraldehyde-3-phosphate dehydrogenase | Mm99999915_g1 |
| Il-6 | interleukin 6 | Mm00446190_m1 |
| Tnf | tumor necrosis factor | Mm00443258_m1 |
| Ugt1a1 | UDP glucuronosyltransferase 1 family, polypeptide A1 | Mm07306663_mH |
| Ugt1a5 | UDP glucuronosyltransferase 1 family, polypeptide A5 | Mm02376089_s1 |

**Table S2.**  Estimates of population pharmacokinetic parameters for olanzapine after p.o. and i.v. administration of olanzapine and, for DMO after i.v. administration of DMO

| Parameter | Olanzapine p.o., i.v. | DMO i.v. |
| --- | --- | --- |
| F_p.o. olanzapine_ | 0.075 (12%) | na |
| βF_LPS | 1.14 (14%) | na |
| k_a_ (hour^−1^) | 2.9 (48%) | na |
| CL (L·hour^−1^.kg^-1^) | 2.84 (8.7%) | 0.62 (19%) |
| βCL_LPS | -0.43 (42%) | -1.54 (21%) |
| Q (L·hour^−1^.kg^-1^) | 9.0 (31%) | 2.51 (30%) |
| V_1_ (L.kg^-1^) | 4.1 (13%) | 1.6 (14%) |
| βV_1__LPS | ns | -1.2 (15%) |
| V_2_ (L.kg^-1^) | 3.5 (18%) | 1.49 (14%) |
| βV_2__LPS | ns | -0.56 (38%) |
| Percent interindividual variability (%RSE) | |  |
| ωF | 16 (38%) | na |
| ωk_a_ | 145 (30%) | na |
| ωCL | 24 (37%) | 54 (34%) |
| ωQ | 73 (49%) | 17 (90%) |
| ωV_1_ | 45 (32%) | 37 (18%) |
| ωV_2_ | 83 (20%) | 13 (122%) |
| Residual variability |  |  |
| Proportional error | 0.16 (29%) | 0.11 (17%) |

Data are population medians (percent relative standard errors). Abbreviations: na…not aplicable, ns…not significant

**Table S3.**  Results of joint parent-metabolite modelling performed separately in LPS and control groups. Estimates of the rate and extent of presystemic first-pass formation of DMO and, of the first-order rate constant of DMO formation from olanzapine in the plasma.

| Parameter | LPS | Controls |
| --- | --- | --- |
| F_p.o._ (olanzapine+DMO) | 0.25 (13 %) | 0.11 (8.0 %) |
| F_d_ | 0.84 (3.9 %) | 0.73 (2.0 %) |
| k_a_ (hour^−1^) | 2.4 (52 %) | 4.6 (51 %) |
| k_aDMO_ (hour^−1^) | 1.7 (37 %) | 2.0 (38 %) |
| k_pm_ (hour^−1^) | 0.033 (7.6 %) | 0.068 (9.3 %) |
| Interindividual variability (%) |  |  |
| ωF_p.o._ | 24 (52 %) | 14 (79 %) |
| ωF_d_ | 8.2 (78 %) | 6.3 (63 %) |
| ωk_a_ | 230 (32 %) | 51 (65 %) |
| ωk_aDMO_ | 45 (32 %) | 52 (73 %) |
| ωk_pm_ | 29 (34 %) | 14 (82 %) |
| Residual variability |  |  |
| Proportional error olanzapine | 0.26 (17 %) | 0.071 (36 %) |
| Proportional error DMO | 0.15 (21%) | 0.17 (22%) |

Data are population medians (percent relative standard errors).

**Table S4.** Median (interquartile range) values for biochemistry tests.

|  | | LPS mice | Controls | | P-value | |
| --- | --- | --- | --- | --- | --- | --- |
| Interleukin-6 (pg/mL) | | 98 (47-212) | 10.7 (9.7-14) | | <0.001 | |
| Urea (mmol/L) | | 9.8 (7.8-27) | 7.9 (6.7-9.5) | | <0.05 | |
| Creatinine (mmol/L) | | 18 (18-30) | 18 (18-22) | | 0.41 | |
| AST (μcat/L) | | 1.6 (1.1-2.2) | 1.1 (0.9-1.3) | | <0.05 | |
| ALT (μcat/L) | | 0.8 (0.5-1.1) | 0.7 (0.5-0.8) | | 0.42 | |
| ALP (μcat/L) | | 0.7 (0.6-1.1) | 1.1 (1.0-1.2) | | <0.01 | |
| Total bilirubin (μmol/L) | | 5 (5-6) | 5 (5-5) | | 0.15 | |
| Albumin (g/L) | | 27 (25-30) | 33 (32-35) | | <0.001 | |
| Globulins (g/L) | | 19 (17-21) | 12 (11-13) | | <0.001 | |
| Total protein (g/L) | | 46 (44-48) | 45 (44-48) | | 0.42 | |
| Glucose (mmol/L) | | 9.3 (6.8-10.7) | 15.5 (13.9-16.6) | | <0.001 | |
| Na^+^ (mmol/L) | | 151 (149-153) | 149 (148-150) | | 0.15 | |
| K^+^ (mmol/L) | | 5.0 (4.6-5.2) | 5.0 (4.7-5.3) | | 0.71 | |
| Cl^-^ (mmol/L) | | 112 (109-114) | 110 (109-112) | | 0.22 | |
| Ca (mmol/L) | 2.22 (2.15-2.31) | | 2.23 (2.18-2.27) | 0.92 |  |  |

Symbols: AST aspartate aminotransferase, alanine aminotransferase, ALP alkaline phosphatase

**
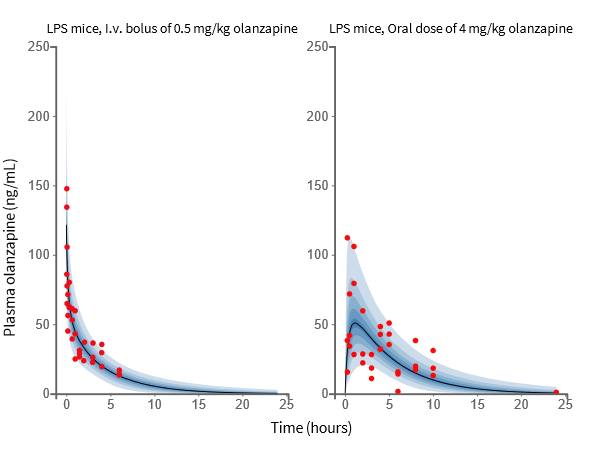
**
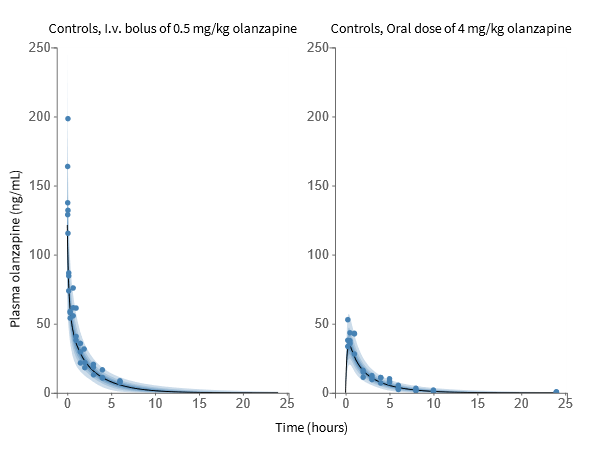
 **Figure S1**. Visual predictive checks of olanzapine concentrations in the plasma after a single i.v. bolus dose of 0.5 mg/kg olanzapine, and after a single oral dose of 4 mg/kg olanzapine. The solid lines are the median predictions from the population model and the shaded areas are the 90% prediction intervals for concentrations. The symbols show the assayed concentrations.


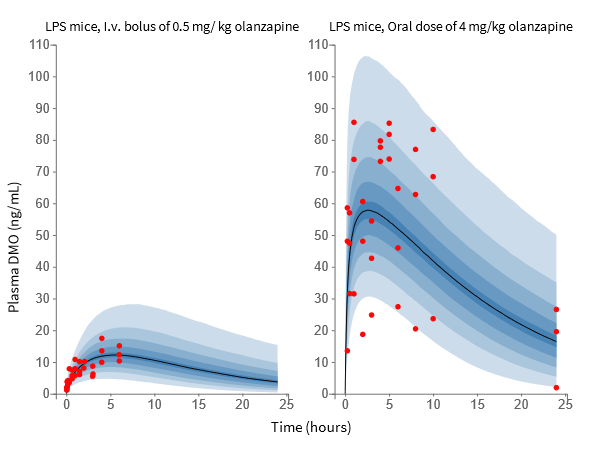

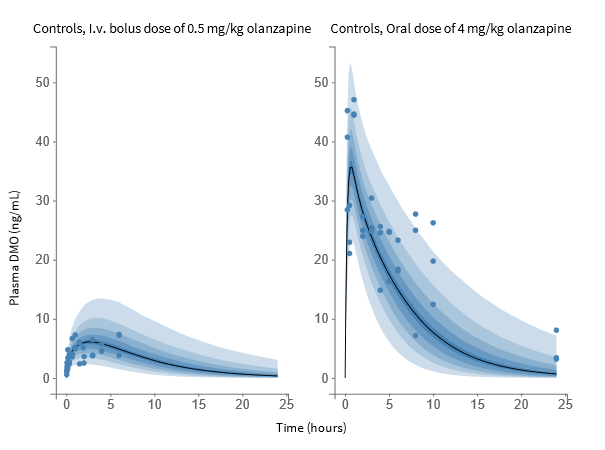


**Figure S2**. Visual predictive checks of DMO concentrations in the plasma after a single i.v. bolus dose of 0.5 mg/kg olanzapine, and after a single oral dose of 4 mg/kg olanzapine. The solid lines are the median predictions from the population model and the shaded areas are the 90% prediction intervals for concentrations. The symbols show the assayed concentrations.


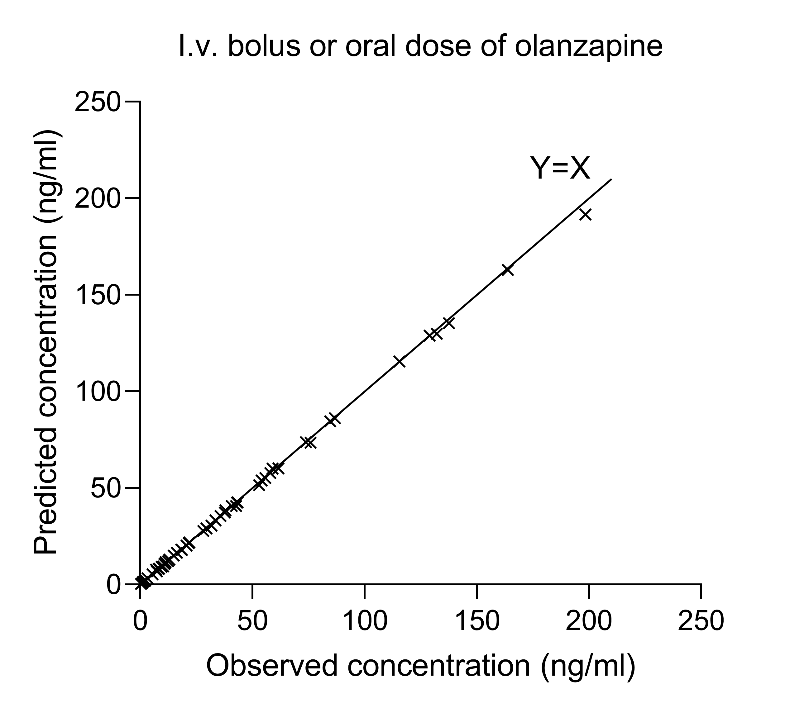


**Figure S3**. Scatter plot of individually predicted plasma concentrations of olanzapine plotted against assayed concentrations.


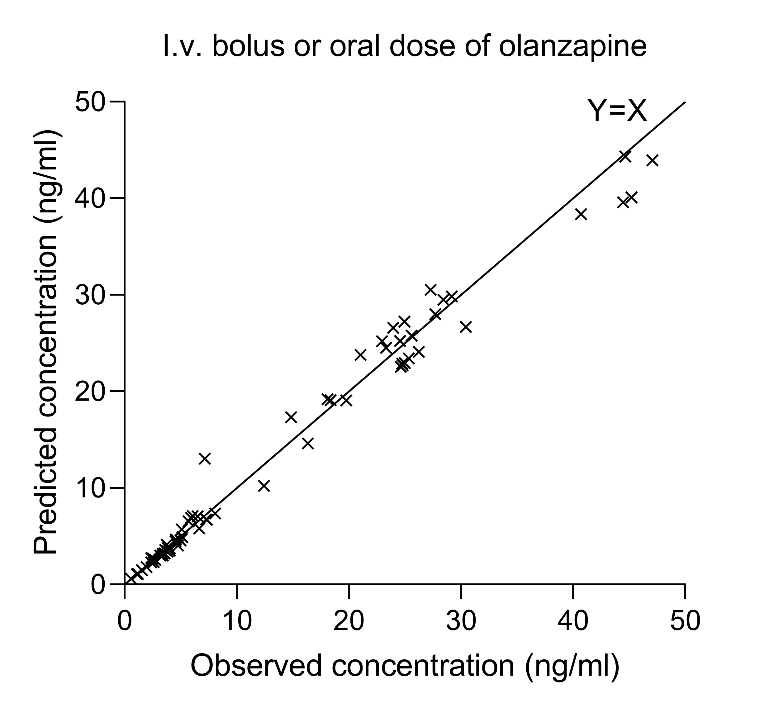


**Figure S4**. Scatter plot of individually predicted plasma concentrations of DMO plotted against assayed concentrations.


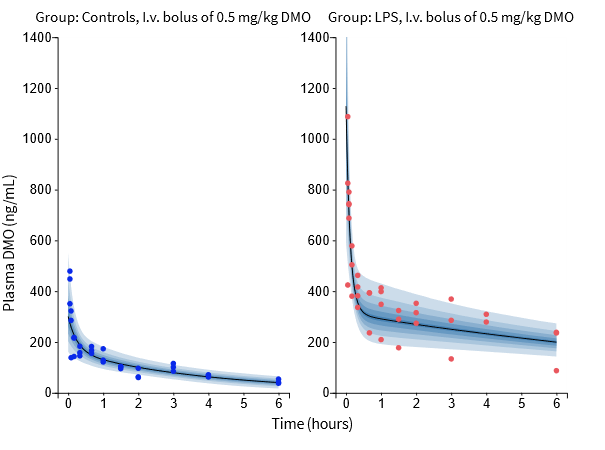


**Figure S5**. Visual predictive checks of DMO concentrations in the plasma after a single i.v. bolus dose of 0.5 mg/kg DMO


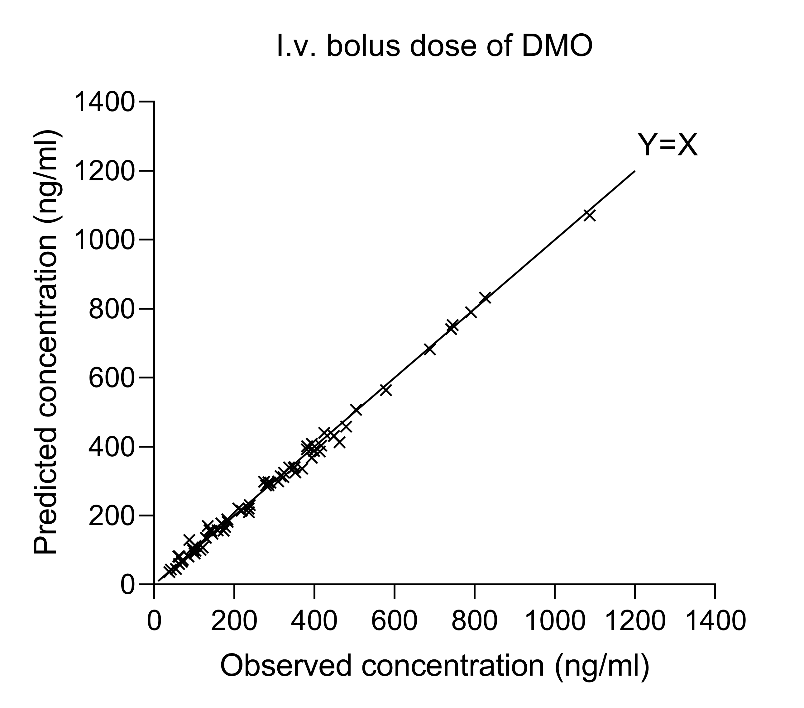


**Figure S6**. Scatter plot of individually predicted plasma concentrations of DMO plotted against assayed concentrations.

**References**

1. Turner PV, Brabb T, Pekow C, Vasbinder MA. Administration of substances to laboratory animals: routes of administration and factors to consider. *J Am Assoc Lab Anim Sci.* 2011;50:600-613.
2. Thomas S. Kaye, Merrill J. Egorin, Charles E. Riggs, Eve A. Olman, Feng-te E. Chou, Michael Salcman, The plasma pharmacokinetics and tissue distribution of dimethyl sulfoxide in mice. *Life Sci*. 1983;33:1223-1230.
3. McConnell EL, Basit AW, Murdan S. Measurements of rat and mouse gastrointestinal pH, fluid and lymphoid tissue, and implications for in-vivo experiments. *J Pharm Pharmacol.* 2008;60:63-70.
4. Gad SC, Spainhour CB, Shoemake C, Pallman DR, Stricker-Krongrad A, Downing PA, Seals RE, Eagle LA, Polhamus K, Daly J. Tolerable Levels of Nonclinical Vehicles and Formulations Used in Studies by Multiple Routes in Multiple Species With Notes on Methods to Improve Utility. *Int J Toxicol.* 2016;35:95-178.
5. Gopinathan S, O'Neill E, Rodriguez LA, Champ R, Phillips M, Nouraldeen A, Wendt M, Wilson AGE, Kramer JA. In vivo toxicology of excipients commonly employed in drug discovery in rats. *J Pharmacol Toxicol Methods.* 2013;68:284-295.
6. Watanabe E, Sudo R, Takahashi M, Hayashi M. Evaluation of absorbability of poorly water-soluble drugs: validity of the use of additives. *Biol Pharm Bull.* 2000;23:838-843.
7. O'Shea JP, Augustijns P, Brandl M, Brayden DJ, Brouwers J, Griffin BT, Holm R, Jacobsen AC, Lennernäs H, Vinarov Z, O'Driscoll CM. Best practices in current models mimicking drug permeability in the gastrointestinal tract - An UNGAP review. *Eur J Pharm Sci.* 2022;170:106098.
8. Hamid KA, Katsumi H, Sakane T, Yamamoto A. The effects of common solubilizing agents.

*Int J Pharm*. 2009;379:100-108.

1. Tanaka Y, Kubota A, Matsuo A, Kawakami A, Kamizi H, Mochigoe A, Hiramachi T, Kasaoka S, Yoshikawa H, Nagata S. Effect of Absorption Behavior of Solubilizers on Drug Dissolution in the Gastrointestinal Tract: Evaluation Based on In Vivo Luminal Concentration-Time Profile of Cilostazol, a Poorly Soluble Drug, and Solubilizers. *J Pharm Sci.* 2016;105:2825-2831
2. Chang CK, Albarillo MV, SchumerW. Therapeutic effect of dimethyl sulfoxide on ICAM-1 gene expression and activation of NF-kB and AP-1 in septic rats. *J Surg Res.* 2001;95:181–187.
3. Kelly KA, Hill MR, Youkhana K, Wanker F, Gimble JM. Dimethyl sulfoxide modulates NF-kB and cytokine activation in lipopolysaccharide-treated murine macrophages. *Infect Immun.* 1994;62:3122–3128
4. Li D, Han Y, Meng X, Sun X, Yu Q, Li Y, Wan L, Huo Y, Guo C.Effect of regular organic solvents on cytochrome P450-mediated metabolic activities in rat liver microsomes. *Drug Metab Dispos.* 2010;38:1922-1925.
5. Iyer KR, Kamble SH, Shah TS, Patil P, Tomar TS, Betgiri S, Tanna RS, Sankaran S and Raikuvar K. In vitro studies on the effects of water miscible organic co-solvents on select drug metabolizing enzyme activities. *Indian J Pharm Sci.* 2024;86:546-555.
6. Uchaipichat V, Mackenzie PI, Guo XH et al. Human UDP-glucuronosyltransferases: isoform selectivity and kinetics of 4-methylumbelliferone and 1-naphthol glucuronidation, effects of organic solvents, and inhibition by diclofenac and probenecid. *Drug Metab Dispos.* 2004;32:413–423.
7. Ruiz-Picazo A, Gonzalez-Alvarez M, Gonzalez-Alvarez I, Bermejo M. Effect of Common Excipients on Intestinal Drug Absorption in Wistar Rat. Molecular Pharmaceutics 2020; 17 :2310-2318.
8. (Chassaing B., Koren O., Goodrich J.K., Poole A.C., Srinivasan S., Ley R.E., Gewirtz A.T. Dietary emulsifiers impact the mouse gut microbiota promoting colitis and metabolic syndrome. Nature. 2015;519:92–96).
9. 1/ EMA, Guideline on Bioanalytical Method Validation, 21 July 2011 (Accessed 4 December 2023) [http://www.ema.europa.eu/docs/enGB/documentlibrary/Scientific guideline/2011/08/WC500109686.pdf](http://www.ema.europa.eu/docs/enGB/documentlibrary/Scientific%20guideline/2011/08/WC500109686.pdf)
10. 2/FDA, Guidance for Industry, Bioanalytical Method Validation, Center for Drug Evaluation and Research (CDER). Center for Veterinary Medicine (CVM), 2018 (Accessed 4 December 2023) <https://www.fda.gov/downloads/Drugs/Guidance/ucm070107.pdf>
